# Pseudouridine synthases are proviral factors for Sindbis virus in insect and mammalian cells

**DOI:** 10.1128/mbio.01329-25

**Published:** 2025-05-27

**Authors:** Nicole Stark, Ram Podicheti, Lauren Garcia, Adela Krenz, Douglas B. Rusch, Irene L. G. Newton, Richard W. Hardy

**Affiliations:** 1 Department of Biology, Indiana University 123993, Bloomington, Indiana, USA; 2 Center for Genomics and Bioinformatics, Indiana University 151786, Bloomington, Indiana, USA; Washington University in St. Louis School of Medicine, St. Louis, Missouri, USA

**Keywords:** alphavirus, pseudouridine, proviral

## Abstract

Alphaviruses are positive-sense, single-stranded RNA (+ssRNA) viruses transmitted by arthropod vectors to vertebrate hosts. Pseudouridine is the most prevalent RNA modification in the prototype alphavirus, Sindbis virus (SINV) genome, but the location, function, and the cellular machinery that deposits pseudouridine are not known. Here, we demonstrate that the host pseudouridine synthase, Nop60B, plays a proviral role in SINV replication in *Drosophila melanogaster*. We show that SINV infection alters the expression of *Nop60B* isoforms and that SINV RNA levels significantly correlate with *Nop60B* RNA levels in infected flies. Furthermore, ectopic expression of Nop60B in cell culture increased SINV infectivity and intracellular RNA levels, which is dependent on the catalytic function of Nop60B. We found that this proviral function is conserved as the human ortholog, dyskerin, increases SINV replication. Using Psi-seq, we mapped putative pseudouridine sites within SINV RNA. Most of the putative psi sites identified were in the structural protein coding region, and we identified a stretch of putative pseudouridine residues surrounded by sequences complementary to a snoRNA known to guide Nop60B to its RNA target. A silent mutation at one of these sites led to a reduction in SINV replication, indicating a potential functional role. Overall, our findings suggest that pseudouridine and the pseudouridine synthases contribute to alphavirus replication, adding to our broader understanding of viral replication mechanisms.

**IMPORTANCE:**

Alphaviruses pose a threat to over half of the global population, and currently, there are no approved antivirals targeting alphaviruses. We identified a conserved pseudouridine synthase that is proviral for Sindbis virus (SINV) infection in insects and humans. Using Psi-seq, we identified putative pseudouridine residues in SINV RNA. Mutagenesis of putative psi sites led to a slight reduction in replication and suggests that pseudouridine residues in SINV RNA are functionally important in replication.

## INTRODUCTION

Alphaviruses are positive-sense, single-stranded RNA (ssRNA) viruses transmitted between vertebrate hosts and arthropod vectors, most commonly mosquitoes (1). This genus includes several pathogens such as Chikungunya virus (CHIKV), Ross River virus, and the Eastern, Western, and Venezuelan equine encephalitis viruses. Less pathogenic viruses include Sindbis virus (SINV) and Semliki Forest virus. In humans, symptoms from alphavirus infections range from fever, rash, severe muscle, and joint pain to fatal encephalitis. CHIKV has caused multiple outbreaks around the Indian Ocean and has since spread to parts of the Americas (2, 3). Emerging or re-emerging alphavirus infections in human populations are expected to rise due to the adaptation to mosquito vectors and the expansion of vector ranges (2–4). A deeper understanding of alphavirus replication is crucial for the development of effective anti-viral therapies.

Alphavirus genomes encode proteins essential for replication and particle assembly. The genome, approximately 11.7 kb in length, is packaged inside the virus particle, which consists of a nucleocapsid and host-derived lipid membrane with embedded viral glycoproteins (1, 5). Upon entry into the cell, the viral genome is released from the particle and translated (1). The genome has two open reading frames (ORFs). The first ORF, translated from genomic RNA, encodes a polyprotein that is proteolytically processed, producing the four individual non-structural proteins required for alphavirus RNA synthesis (6). A minus-strand RNA intermediate is produced during replication and serves as a template for the synthesis of both genomic RNA and subgenomic RNA (7). The subgenomic RNA is synthesized from an internal promoter within the minus strand. The subgenomic RNA is co-linear with the 3′ end of the genomic RNA and contains the second ORF, which encodes structural proteins that form the virus particle. The newly synthesized genomic RNAs are encapsidated and released from host cells (7).

The transmission cycles of alphaviruses involve multiple host species and multiple tissues within each host. This makes it crucial for them to optimize their genome’s functionality, allowing adaptation to different host and cellular environments. One possible adaptation includes RNA modifications, as increasing evidence indicates that post-transcriptional RNA modifications in RNA virus genomes have a regulatory role in infection. N6-methyladenosine (m^6^A) negatively regulates hepatitis C virus infectious particle production (8). Furthermore, SINV acquires 5-methylcytosine (m^5^C) modifications in mosquito and mammalian cells (9, 10). We have shown that DNMT2, a conserved host m^5^C methyltransferase, enhances the infectivity of SINV produced in mosquito cells, which also correlates with an increase in m^5^C modifications in genomic RNA (9). Multiple studies show that various RNA modifications are present on positive-sense RNA viral genomes, and notably, pseudouridine is the most abundant modification found (10, 11). In cellular RNA, pseudouridine residues alter RNA stability and structure (12, 13), which can have impacts on translation, splicing, and immunogenicity (14–17). However, the role of pseudouridine in alphavirus replication is not known.

Pseudouridine synthases convert uridine to pseudouridine. Given the prevalence of pseudouridine in SINV (10), we began investigating different pseudouridine synthases in *Drosophila melanogaster* to determine a potential role in alphavirus replication. We chose *D. melanogaster* as a model organism given that it is a model for mosquito insect immunity (18), its susceptibility to the prototype alphavirus, SINV, and powerful genetic tools. Of the nine proteins with an annotated pseudouridine synthase domain, *Nop60B* was shown to be highly upregulated due to a SINV replicon (19). Nop60B is the catalytic subunit of an H/ACA ribonucleoprotein (RNP) that forms a complex with three auxiliary proteins (Gar1, NHP2, and Nop10) and a snoRNA that acts as a scaffold (20, 21). The snoRNA guides the RNP complex to the target RNA for pseudouridylation; this is done through base pairing with the substrate RNA (22). H/ACA RNP complexes are highly conserved from archaea to eukaryotes and have an essential role in ribosomal RNA processing (21, 23).

In this study, we identified *Nop60B* as a host factor that enhances SINV replication in *D. melanogaster*. We employed a transgenic RNAi fly line to investigate the consequence of variation in *Nop60B* gene expression on virus replication, finding that *Nop60B* levels positively correlate with SINV RNA levels. Using *D. melanogaster*-derived cell lines to confirm these results, we showed that Nop60B is a proviral host factor increasing intracellular SINV RNA levels and particle infectivity. We investigated the human ortholog, dyskerin, and found that overexpression is proviral for SINV in human cells. We hypothesized that the proviral activity of Nop60B and dyskerin might involve the direct modification of viral RNA. Therefore, we mapped putative pseudouridine residues in SINV RNA using Psi-seq in *Drosophila* cells. Silent mutagenesis of a putative psi site in E2 resulted in a subtle growth defect phenotype relative to wild-type virus in arthropod and mammalian cells. In sum, our results highlight the importance of another RNA modification—pseudouridylation—to the virus life cycle.

## RESULTS

### SINV infection influences *Nop60B* isoform distribution

**Fig 1:**
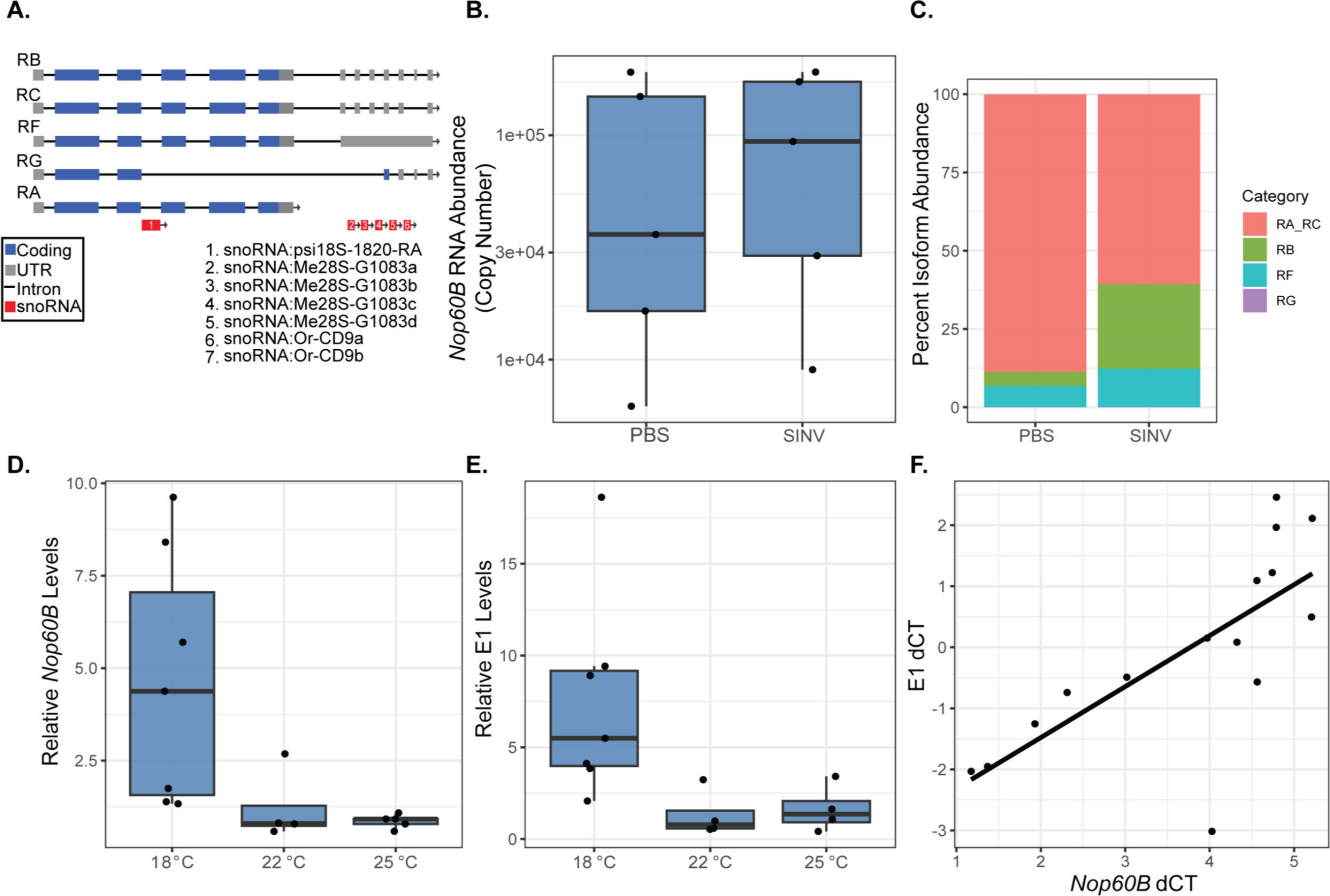
(**A**) Schematic representation of *Nop60B* isoforms RB, RC, RF, RG, and RA. Coding regions (blue), untranslated region (gray), introns (black), and snoRNA regions (red). (**B**) *Nop60B* abundance measured 2 days after injection in adult female flies using qRT-PCR with primers designed to target all isoforms. Each replicate represents an individual fly. (**C**) Percent isoform abundance of *Nop60B* isoforms within samples from (B). Percent isoform abundance was determined by averaging the total RNA abundance of each isoform across all samples within each group, then by dividing by the average total isoform abundance and multiplying by 100. (**D**) Relative *Nop60B* levels in flies reared at 18°C, 22°C, and 25°C measured using qRT-PCR. Primers targeted all Nop60B isoforms, and data were normalized to *Rpl32*. (**E**) Relative E1 RNA levels in flies from (**D**). (**F**) Linear regression of E1 dCT values and Nop60B dCT values. Pearson’s *r*, *P* = 0.0020, *r* = 0.6619.

We initially chose to characterize how *Nop60B* RNA levels respond to SINV infection in whole flies. Wild-type flies were injected with PBS or SINV and collected 2 days post-injection. No significant changes in *Nop60B* expression across the samples were detected using primers targeting all *Nop60B* transcript isoforms (Fig. 1A and B). The gene structure of *Nop60B* is complex, as it produces five isoform variants, leading to two protein products. The isoforms mainly differ at the 3′ UTR, and differential splicing can lead to the release of small nucleolar RNAs (snoRNAs) housed in *Nop60B* introns. In previous work, it has been shown that the intracellular bacterium, *Wolbachia pipientis*, which is known to inhibit viral replication in insects, significantly alters isoform usage of *Nop60B* in female flies compared to *Wolbachia*-free flies (24). The presence of *Wolbachia* negatively impacts SINV replication, and this led us to hypothesize that the virus may also influence isoform usage. To examine this possibility, absolute *Nop60B* RNA abundance was measured using primers targeting all *Nop60B* isoforms, and isoforms RG, RB, and RF were also quantified using specific primers (Table S1). Isoforms RA and RC could not be differentiated due to their similarities with other isoforms. To produce an approximate absolute abundance of RA + RC in each sample, we subtracted the absolute abundances of RG, RB, and RF from the absolute abundance of all isoforms. RA + RC pool represented a majority of the isoforms across both groups (Fig. 1C). RA + RC represent 88.68% in PBS-injected flies. SINV infection decreases the RA + RC pool to 60.7% (Fig. 1C).

Furthermore, isoform RB increased to 26.96% due to SINV infection compared to 4.52% in PBS-injected flies (Fig. 1C). SINV infection increases the percentage of the RF isoform by ~2-fold compared to their PBS-injected counterparts (Fig. 1C). RG, which is the isoform encoding for the truncated Nop60B protein product, represented less than 0.05% of each group (Fig. 1C). Overall, the presence of SINV appears to influence *Nop60B* transcript isoform production, suggesting that *Nop60B* influences virus replication. We examined the gene expression of seven additional pseudouridine synthases in response to SINV infection. We found no significant change in expression in the other pseudouridine synthases, except for *CG3045*, which was significantly downregulated in SINV flies compared to PBS controls (Fig. S1A through G).

### *Nop60B* RNA and SINV RNA positively correlate in whole flies

Given that SINV infection alters *Nop60B* isoform distribution in wild-type flies, we hypothesized that *Nop60B* may be a host factor involved in SINV replication. To test this hypothesis, we designed an experiment to create varying levels of *Nop60B* gene expression and examine its correlation with viral replication. Homozygous unmated females from a transgenic RNAi fly line containing a short hairpin targeting *Nop60B* were crossed with homozygous Hsp70:Gal4 males. When exposed to heat, the *Nop60B*-targeting shRNA should be expressed and knock down all *Nop60B* isoforms. However, the HSP70:Gal4 heat shock driver is leaky even at room temperature (22°C)*,* making it difficult to consistently control the level of knockdown. Therefore, we reared flies at 18°C, 22°C, and 25°C to induce variation in *Nop60B* expression. *Nop60B* expression and intracellular SINV RNA in individual female flies were measured by qRT-PCR. Overall, *Nop60B* expression decreased as rearing temperature increased, confirming that higher temperatures led to increased expression of the shRNA, which in turn generated a gradient of *Nop60B* RNA levels (Fig. 1D). Intracellular virus RNA decreased at the higher temperatures (22°C and 25°C) relative to 18°C (Fig. 1E). Analysis of data by linear regression found a significant positive correlation between SINV RNA and *Nop60B* RNA levels (Pearson’s *r* = 0.6619, *P* = 0.0020) (Fig. 1F). These results imply that *Nop60B* is a proviral host factor, as more *Nop60B* RNA leads to higher levels of viral RNA. To control for effects on virus replication due to temperature, SINV was injected into homozygous parental transgenic female flies to determine the correlation between *Nop60B* RNA and intracellular SINV E1 RNA. No significant correlation was found between *Nop60B* and E1 in the absence of the driver (Fig. S2).

We next examined whether reduced Nop60B RNA levels affect virus replication in cell culture. We used dsRNA against all *Nop60B* transcripts to knock down gene expression in JW18-TET cells. Cells were given *Nop60B*-specific or non-targeting (NT) dsRNA as a control. Two days post-dsRNA introduction, we infected with SINV TE12 Capsid-mCherry at an MOI of 1. We used live-cell imaging to track virus spread over time and collected final time points to determine virus infectivity. We observed a moderate twofold decrease in *Nop60B* RNA relative to NT dsRNA-treated cells (Fig. S3A). Four days post-infection, we identified nearly a 10% decrease in virus spread in *Nop60B* knockdown cells compared to the control (ANOVA: Time × Nop60B dsRNA, *F*_(8,64)_ = 8.835, *P* < 0.0001; Time, *F*_(1.138,9.100)_ = 621.0, *P* < 0.0001; Nop60B dsRNA, *F*_(1, 8)_ = 8.881, *P* = 0.0176). We collected end-point samples to determine viral titers. We observed about a 1.55-fold decrease in viral titers in virus produced in cells given *Nop60B*-targeting dsRNA compared to the control (Fig. S3B and C), further supporting the results obtained in the animal model that Nop60B is a proviral host factor.

### Ectopic expression of Nop60B is proviral in cell culture

**Fig 2:**
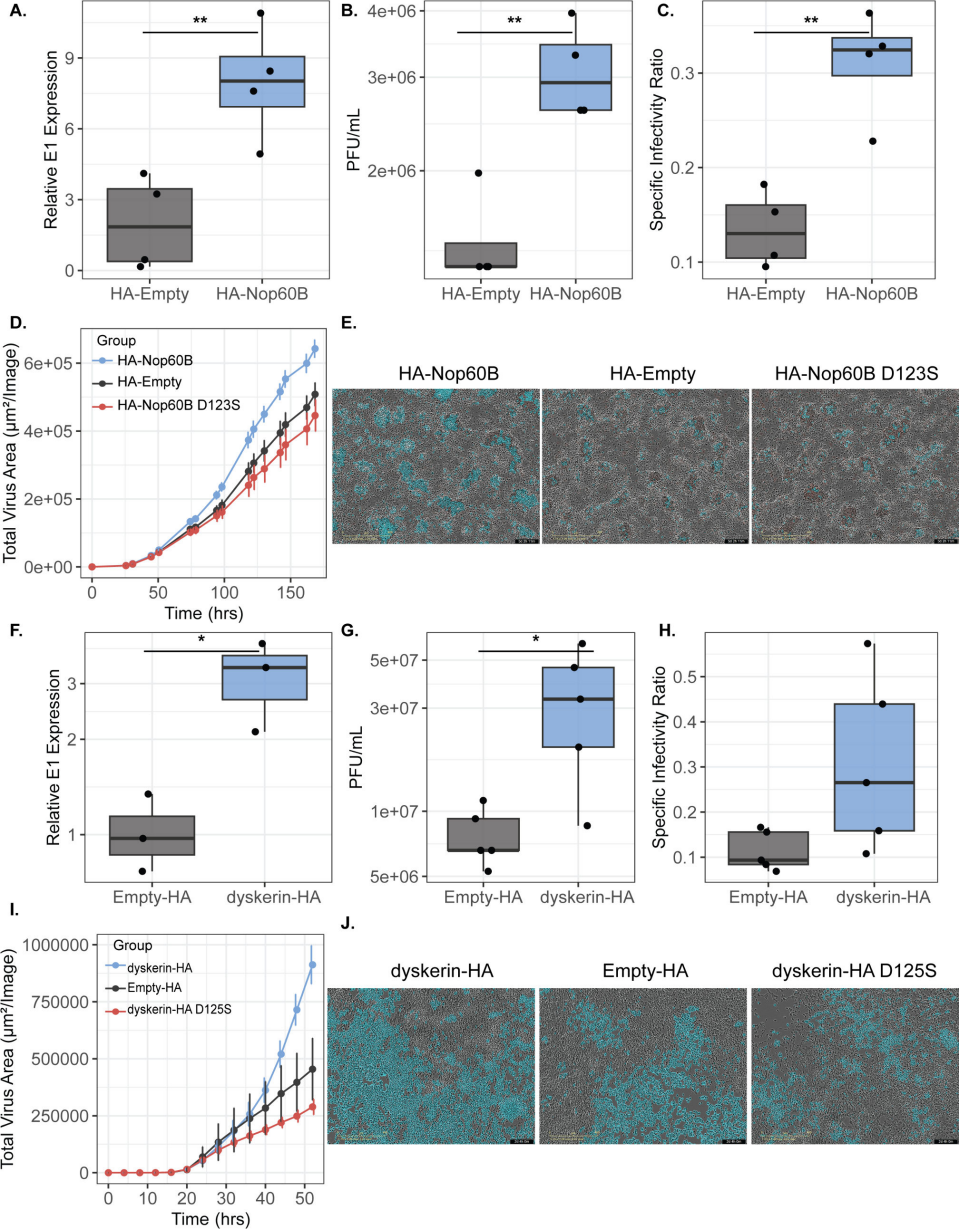
(**A**) S2R+ cells were transfected with expression vectors HA-Nop60B or HA-Empty for 48 h prior to infection with SINV at an MOI of 10. Relative intracellular SINV RNA levels were quantified using qRT-PCR, and data were normalized to actin and expressed as fold change (unpaired, Student’s *t*-test *P* = 0.0091, *t* = 3.787, df = 6). (**B**) Infectious progeny (PFU/mL) produced from these cells were determined through plaque assays on BHK-21 cells (unpaired, Student’s *t*-test *P* = 0.0043, *t* = 4.456, df = 6). (**C**) Specific infectivity ratios of progeny viruses were calculated as the ratio of infectious virus to total virus (genome copies present in supernatant using qRT-PCR) (unpaired, Student’s *t*-test *P* = 0.0025, *t* = 4.974, df = 6). (**D**) Total virus area (µm^2^/Image) over time tracking infection of SINV TE12 tagged with mCherry in S2R+ cells expressing HA-Empty vector (black), HA-Nop60B (light blue), and HA-Nop60B D123S (red). Statistical significance was assessed by two-way ANOVA with Tukey’s post hoc for multivariate analyses. Error bars represent standard error of mean (SEM) of independent experimental replicates (*n* = 6). Next, we overexpressed dyskerin-HA in HEK293T and infected with SINV TE12 tagged with mCherry (MOI = 0.1). (**E**) Representative Incucyte images from infections in panel (**D**). Phase and red channels were used for imaging. Blue represents the analysis mask overlaid on the image, indicating that the region contains fluorescence surpassing the intensity thresholds. Scale bars represent 400 µm. (**F**) Intracellular SINV RNA by qRT-PCR, and data were normalized to GAPDH and expressed as fold change. (**G**) Infectious virus (PFU/mL) was collected 52 h post-infection and determined through plaque assay on BHK-21 cells. (**H**) Specific infectivity ratios were calculated as ratios of infectious virus to total virus genomes. (**I**) Total virus area (µm^2^/Image) over time tracking infection of SINV TE12 tagged with mCherry in HEK293T cells with dyskerin-HA (light blue) (*n* = 5), HA-empty control (black) (*n* = 5), and dyskerin-HA D125S (red) (*n* = 5). (**F**) Representative Incucyte images from infections in panel (**I**). Phase and red channels were used for imaging. Statistical significance was assessed by two-way ANOVA with Tukey’s post hoc for multivariate analyses coupled with *t*-test for pairwise analyses. Error bars represent the standard error of the mean (SEM) of independent experimental replicates. For all panels: ***P < 0.001; **P < 0.01; and *P < 0.05.

Given that *Nop60B* RNA levels and SINV RNA positively correlate, we reasoned that ectopic expression of Nop60B tagged with an HA epitope (HA-Nop60B) would lead to an increase in SINV replication in cell culture. S2R+ cells were transfected with HA-Nop60B and a control vector (HA-Empty) for 48 h before infecting cells at an MOI of 10. We confirmed ectopic expression through western blot (Fig. S4A). After 48 h post-infection, we observed approximately a fourfold increase in intracellular SINV RNA in cells expressing HA-Nop60B compared to control cells (*t*-test: *P* = 0.0091) (Fig. 2A), which corresponds to a threefold increase in virus titer (*t*-test: *P* = 0.0043) (Fig. 2B). Previous work has shown that modification of viral RNA leads to increased infectivity of progeny virus (9); therefore, we examined the effect of *Nop60B* expression on specific infectivity by measuring the ratio of infectious virus to viral genome copies present in the supernatant. We found that overexpressing Nop60B significantly increased progeny virus-specific infectivity (*t*-test: *P* = 0.0025) (Fig. 2C). Similar results were obtained when we examined HA-Nop60B in JW18-TET cells; however, the magnitude of the effect was smaller, likely due to a decreased efficiency of HA-Nop60B transfection in JW18-TET cells (Fig. S4B through F).

To determine if the catalytic activity of Nop60B is responsible for the proviral phenotype, we generated a catalytic mutant (HA-Nop60B D123S). S2R+ cells were transfected with plasmid expressing Nop60B, Nop60B-D123S, and control plasmid, then infected with SINV TE12 Capsid-mCherry at an MOI of 5. Infection was monitored by live-cell imaging using fluorescent protein expression as a proxy for infection. As anticipated, HA-Nop60B significantly increased virus replication over time relative to the empty vector control (ANOVA: Nop60B, *F*_1,10_ = 7.644, *P* = 0.0200; Time, *F*_1.092, 10.92_ = 455, *P* < 0.0001; Time × Nop60B, *F*_15,150_ = 7.988, *P* < 0.0001) (Fig. 2D and E). There was no statistically significant difference in virus replication between the catalytic mutant (Nop60B D123S) and empty vector, suggesting that the catalytic activity of Nop60B is necessary for the proviral phenotype (ANOVA: Nop60B D123S, *F*_1,10_ = 0.8940, *P* = 0.3667; Time, *F*_1.014, 10.14_ = 186.7, *P* < 0.0001; Time × Nop60B D123S, *F*_15,150_ = 1.158, *P* = 0.3111) (Fig. 2D and E). Importantly, ectopic expression of all three constructs showed no significant changes in cell growth (Fig. S2G). In summary, the evidence gathered suggests that the catalytic activity of Nop60B promotes SINV replication in *D. melanogaster*.

Nop60B is evolutionarily conserved with orthologs in multiple organisms, including humans.

We hypothesized that the human ortholog of Nop60B, dyskerin, would also play a proviral role in SINV replication. We ectopically expressed dyskerin fused to an HA tag at the C-terminus (dyskerin-HA) in human HEK293T cells as confirmed through western blot (Fig. S4H). Given that there is a commercially available antibody for dyskerin, we compared ectopically expressed dyskerin levels to those of the empty vector and a no transfection (NT) control. We identified a 3.42× and 4× increase in dyskerin-HA samples compared to empty-HA and the NT control, respectively (Fig. S4J). Next, we infected cells at an MOI of 0.1 2 days post-transfection. Following dyskerin-HA overexpression, we observed about a threefold increase in SINV intracellular RNA in the dyskerin-HA relative to the control 52 h post-infection (Fig. 2F) (*t*-test, *P* = 0.0214). Dyskerin-HA overexpression experiments led to a half log increase in virus titer (2G) (*t*-test, *P* = 0.0472). Additionally, we found that overexpressing dyskerin-HA increased the specific infectivity ratio; however, the increase was not statistically significant (Fig. 2H) (*t*-test, *P* = 0.0878). Next, we generated a dyskerin catalytic mutant (dyskerin-HA D125S) to determine if the catalytic activity of dyskerin is essential for its proviral phenotype. To assess the effects on virus replication, we transfected HEK293T cells and infected them with SINV (MOI = 0.1) 2 days post-transfection. Using a fluorescent marker as a proxy for virus replication, we observed a significant increase in replication due to dyskerin-HA ectopic expression relative to the control over time (ANOVA: Time × dyskerin, *F*_(15,120)_ = 7.926, *P* < 0.0001; dyskerin, *F*_(1,8)_ = 3.113, *P* = 0.1157; Time, *F*_(15, 120)_ = 59.96, *P* < 0001) (Fig. 2I and J). Similar to the Nop60B catalytic mutant, we observed no statistically significant difference in replication between the catalytic mutant and the empty vector control (ANOVA: Time × dyskerin D125S, *F*_(15, 120)_ = 1.188, *P* = 0.2904; dyskerin D125S, *F*_(1,8)_ = 0.8066, *P* = 0.3953; Time, F_(1.069, 8.551)_ = 23.02, *P* = 0.0010) (Fig. 2I and J). Ectopic expression of the dyskerin constructs did not change cell growth relative to the empty vector control (Fig. S4H). Overall, these results show that the human ortholog of Nop60B, dyskerin, is also a proviral host factor in SINV replication in human cells.

### Components of the H/ACA RNP complex localize in nuclear and cytoplasmic fractions

It is possible that Nop60B enhances SINV replication by directly targeting the virus genome, or indirectly, through targeting another host factor important in virus replication. Nop60B primarily localizes in the nucleolus, while SINV replication occurs in the cytoplasm. We hypothesized that if Nop60B directly targeted the virus genome, Nop60B would also localize in the cytoplasm. Additionally, since virus replication can alter nuclear-cytoplasmic shuttling (25), we investigated how virus infection affects the localization of HA-Nop60B. We transfected cells with HA-Nop60B or HA-Empty and either mock-treated or infected cells with SINV at an MOI of 5. We first optimized a nuclear/cytoplasmic fractionation protocol and used Histone H3 and Beta-Actin as control markers to confirm separation (Fig. S5A). We next used an HA-antibody to target HA-Nop60B. We found the majority of HA-Nop60B in the nuclear fraction in both mock and SINV-infected cells (Fig. S5A). However, HA-Nop60B was present in cytoplasmic fractions in mock- and SINV-infected cells, albeit at a lower level than the nuclear fraction. There were no apparent differences between the mock and infected samples (Fig. S3A).

Since snoRNA H1, also known as snoRNA:Ψ18S-1820, is an endogenous component of the H/ACA RNP complex, we examined the localization of this snoRNA. We infected S2R+ cells at an MOI of 5 and collected samples 2 days post-infection. Nuclear and cytoplasmic fractions were obtained as above and subsequently split in half for RNA and protein extractions. Separation of nuclear and cytoplasm fractions was confirmed through western blot (Fig. S3B). Using qRT-PCR, we detected snoRNA H1 in both nuclear and cytoplasm fractions in mock- and SINV-infected samples (Fig. S5C). Like HA-Nop60B localization, we see more snoRNA H1 in the nuclear fractions (Fig. S5C). These results indicate that the H/ACA RNP complex (or components thereof) is found in both the nucleus and the cytoplasm in both the presence and absence of SINV.

### Putative pseudouridine residues in SINV RNA derived from *Drosophila* cells

**Fig 3:**
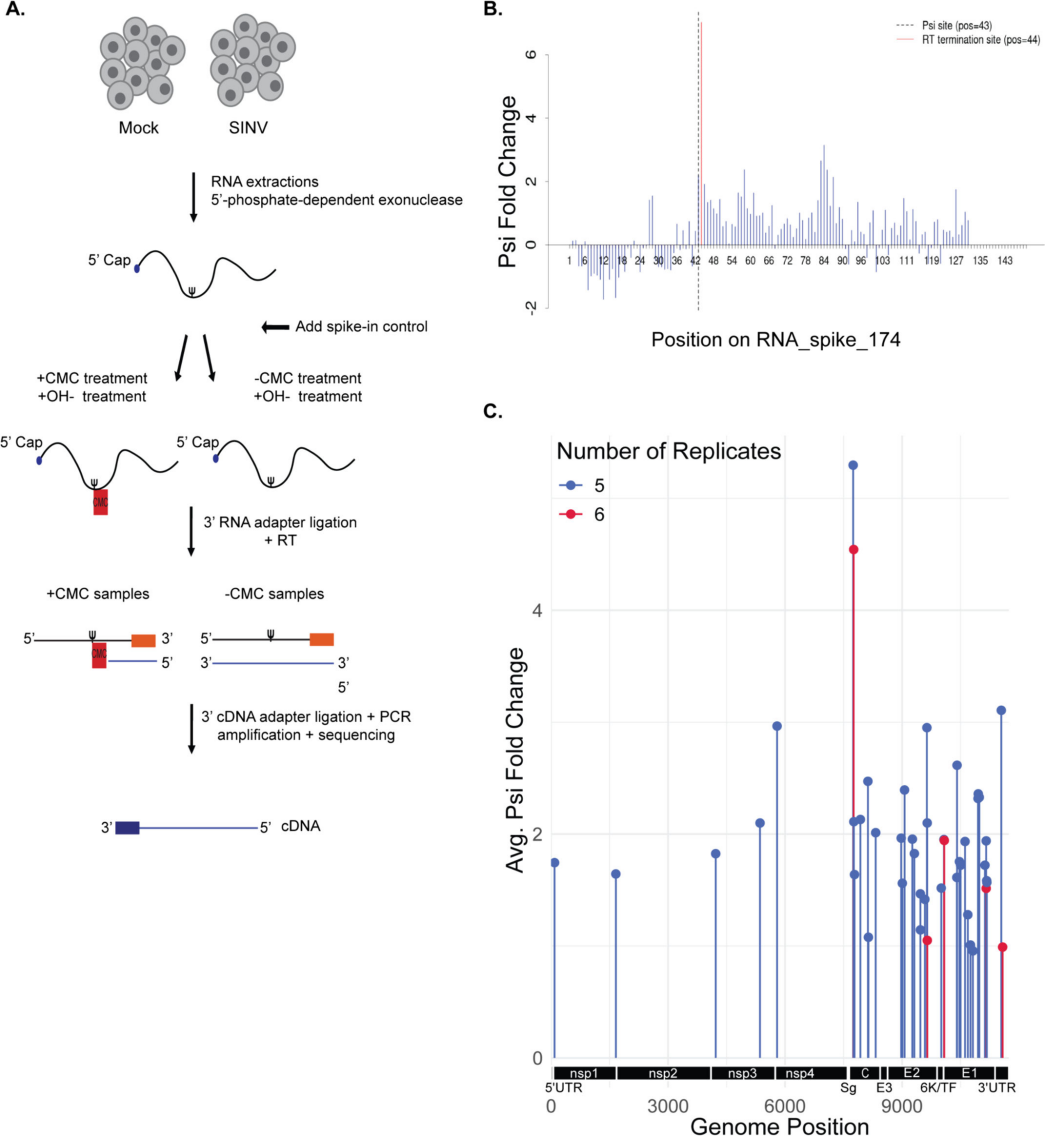
(**A**) Schematic of Psi-seq protocol in JW18-TET cells. Total cellular RNA was extracted from JW18-TET cells. Following ribosomal RNA depletion, we spiked into each sample an internal control molecule containing a single pseudouridine residue at position 43. Samples were split into two before CMC treatments. CMC+ and CMC− libraries were subjected to Illumina library prep. (**B**) Representative graph of the internal control molecule is present in each library, containing a single pseudouridine residue at position 43. The red peak at base 44 is indicative of reverse transcription termination caused by the CMC modification at base 43. (**C**) Average psi fold change (log_2_) of putative psi sites across the virus genome in JW18-TET cells (FDR < 0.0005).

**Fig 4:**
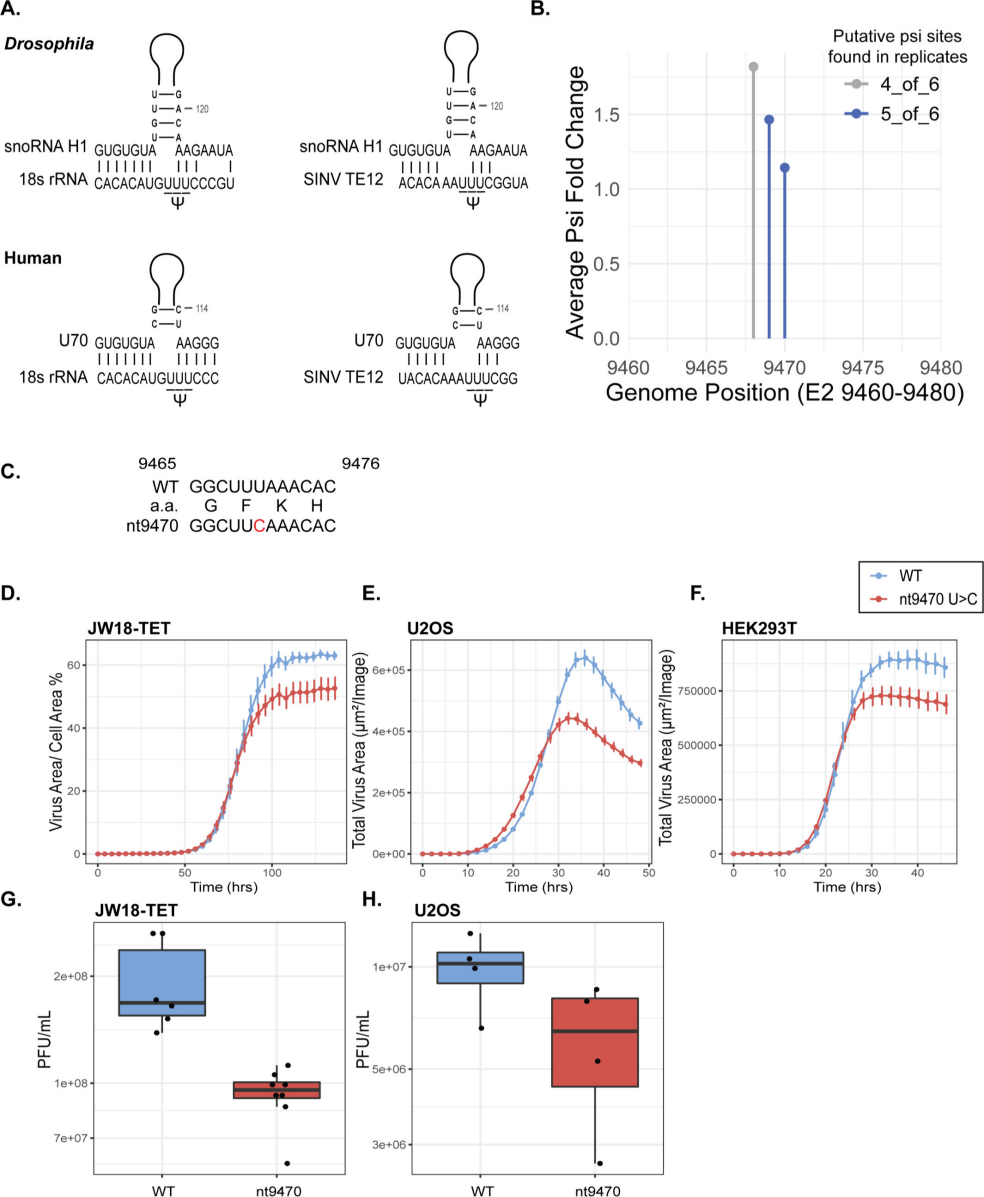
(**A**) Schematic of snoRNA H1 (*Drosophila*) and U70 snoRNA (human) showing complementary base pairing with 18S rRNA and SINV RNA in the E2 gene. (**B**) The average psi fold change of nt9468, 9469, and 9470 in E2. Putative psi sites are indicated in blue (5/6 replicates) and gray (4/6 replicates). (**C**) Nucleotide sequence alignment of wild-type (WT) and nt9470 mutant (U > C), showing corresponding amino acids. (**D**) SINV TE12 and SINV TE12 nt9470 growth over time monitored by live-cell imaging in JW18-TET (*n* = 7), (**E**) U2OS cells (*n* = 5), and (**F**) HEK293T cells (*n* = 9). End-point viral titers of SINV TE12 and SINV TE12 nt9470 in (**G**) JW18-TET cells and (**H**) U2OS cells.

Given the abundance of pseudouridine in SINV RNA (10) and the requirement for its catalytic activity for proviral effect, we reasoned that Nop60B may directly target virus RNA. To explore this hypothesis, we sought to map sites of pseudouridylation within the virus genome using Psi-seq (26). Pseudouridine is read as uridine during RNA sequencing. Therefore, a chemical treatment is needed to distinguish pseudouridine from uridine. N-cyclohexyl-N′-b (4-methylmorpholinium)-ethylcarbodiimide (CMC) covalently binds to pseudouridine, guanosine, and uridine. An alkaline treatment can be applied to remove CMC from guanosine and uridine, leaving CMC bound to pseudouridine residues. As Psi-CMC adducts terminate reverse transcription, CMC treatments have been combined with RNA-seq to map putative pseudouridine residues in cellular RNA (26) (Fig. 3A). We optimized Psi-seq in JW18-TET cells with SINV at an MOI of 10. SINV replication is more productive in this cell line, which is why we chose it over S2R+. Following infections, RNA was extracted and depleted of ribosomal RNA before an internal control molecule was spiked into each sample. Each sample was split into two before CMC+ and CMC− treatments, followed by library preparation for Illumina sequencing. Significant psi-fold change was ensured by examining sequencing termination in the internal control molecule of each sample one base downstream of the pseudouridine residue, indicating that the CMC treatment was effective at terminating the RT reactions (Fig. 3B). We identified 45 putative pseudouridine residues in SINV RNA from JW18-TET cells that were identified in five or more replicates (Fig. 3C; Tables S2 to S8). Most of the putative psi sites were found in structural genes (Fig. 3C). Sequencing identified two putative psi sites in the E2 gene region (nt9469 and nt9470), which is intriguing due to the similarity of this segment to a region in the *D. melanogaster* 18S rRNA where Nop60B catalyzes pseudouridylation of three consecutive U’s (21). Additionally, both the 18S rRNA region and the corresponding SINV RNA segment exhibit complementary base pairing to snoRNA H1 (Fig. 4A). U70 snoRNA is the human equivalent of snoRNA H1 (22) and also has complementary base pairing with SINV RNA surrounding the putative pseudouridine residues identified in E2 (Fig. 4A). Overall, these results support the hypothesis that Nop60B and dyskerin may catalyze pseudouridine modifications at this site in SINV RNA.

### SINV with silent mutation of putative psi residue in E2 exhibits a slight growth defect

RNA modifications in other positive-sense, ssRNA viruses have been shown to affect virus replication (8). We hypothesized that the putative pseudouridine residues in E2 may have functional importance in SINV replication. Nucleotides 9468, 9469, and 9470 all showed significant signal as sites of pseudouridylation, with 9468 being identified in four out of six replicates and 9469 and 9470 in five out of six replicates (Fig. 4B). We opted to change nt9470 as it is the third base in a phenylalanine codon (UUU) and changing it would not result in a coding change; therefore, we generated a silent mutation (U > C) at this site to prevent pseudouridylation (Fig. 4A). To confirm that the mutation at nt9470 prevented pseudouridylation at this site, we did Psi-seq on the mutant. Interestingly, we did not detect pseudouridine at nt9469 or nt9470 (Table 1), suggesting that preventing pseudouridine at one site can affect the modification at another site. To evaluate growth kinetics compared to the parental wild-type SINV TE12-Capsid-mcherry, we infected JW18-TET, U2OS (human), and HEK293T cells at an MOI of 0.1 and monitored virus growth using live-cell imaging. We found that the overall end-point yield of virus replication is slightly reduced in the nt9470 mutant compared to wild-type in arthropod and human cells (Fig. 4C and D; Fig. S6A). Furthermore, the slight reduction in the live-cell imaging data corresponds to a reduction in infectious virus (Fig. 4E and F). Next, we measured the ratio of infectious virus to viral genome copies present in the supernatant. We observed no significant difference in the specific infectivity ratio between WT and nt9470 (Fig. S6B and C), suggesting that an alternative mechanism may be responsible for reduced growth.

**TABLE 1:** Psi-seq results from SINV TE12 WT and SINV TE12 nt9470 derived from JW18-TET cells^*a*^

Position	Metric	WT 1	WT 2	WT 3	nt9470 1	nt9470 2	nt9470 3
9468	Psi fold change	1.3659	1.50046	1.01378	1.62484	1.24695	1.82261
	*Z*-score	5.34435	6.6146	3.59994	3.69148	2.45018	4.48274
	*P* value	4.54E−08	1.86E−11	0.00016	0.00011	0.00714	3.68E−06
	Adj. *P* value (BH)	1.15E−06	2.08E−10	0.00081	0.00079	0.01932	4.57E−05
9469	Psi fold change	1.37043	1.15691	1.00348	.	.	.
	*Z*-score	6.0683	5.50982	4.12226	.	.	.
	*P* value	6.46E−10	1.80E−08	1.88E−05	.	.	.
	Adj. *P* value (BH)	2.49E−08	1.10E−07	0.00013	.	.	.
9470	Psi fold change	0.95731	1.09497	1.10901	0.01635	.	.
	*Z*-score	3.8823	4.88571	4.85098	0.0372	.	.
	*P* value	5.17E−05	5.15E−07	6.14E−07	0.48516	.	.
	Adj. *P* value (BH)	0.00043	2.33E−06	7.81E−06	0.48781	.	.

^*a*^ Empty cells indicate that less than or equal to five termination events occurred, preventing the calculation of a Psi fold change and subsequent statistics.

## DISCUSSION

Over 170 chemical RNA modifications have been reported in RNA species, such as mRNA, rRNA, tRNA, and various other noncoding RNAs (27). RNA modifications, including m^6^A, m^5^C, and pseudouridine, play a regulatory role in stability, transport, splicing, translation, and immunogenicity of RNA molecules (16, 17). While some RNA modifications have been recognized for decades in viral RNA (28, 29), emerging literature indicates that RNA modifications are prevalent and regulate replication in cytoplasmic replicating RNA viruses. Evidence shows m^6^A methyltransferases negatively regulate HCV infectious viral particle production and that m^6^A sites are highly conserved in the Flaviviridae family RNA genomes (8). Moreover, research from our group found that a host m^5^C methyltransferase is a proviral host factor in SINV replication in mosquito cells, in which expression correlates with the amount of m^5^C in SINV particles (9). Pseudouridine was identified in intracellular RNA of various +ssRNA viruses and RNA in particles of SINV from mammalian and mosquito cells (10, 11). However, where pseudouridine residues are in SINV RNA and how pseudouridine modifications affect virus replication remain open questions.

Given that pseudouridine is prevalent in SINV RNA, we began by investigating the potential role of pseudouridine synthases for SINV replication in *D. melanogaster*. We focused on *Nop60B* because, among the nine annotated pseudouridine synthases, it was the only one upregulated in response to a SINV replicon in flies (19). Moreover, in *Wolbachia*-infected flies, a condition where SINV is inhibited, *Wolbachia* alters the transcriptional regulation of *Nop60B* (24). Here, we find that SINV infection in flies alters the isoform distribution of *Nop60B* and that *Nop60B* plays a proviral role in SINV replication in whole flies and cell culture. Given the evolutionary conservation of Nop60B across multiple organisms (30), we investigated the human ortholog, dyskerin. Overexpression of dyskerin in HEK293T cells significantly increased replication of SINV, showing that the proviral phenotype is conserved in humans. Next, we mapped putative pseudouridine residues in SINV RNA from *D. melanogaster* cells using Psi-seq (26). We identified putative pseudouridine residues primarily in genes encoding for structural proteins. Finally, we identified a stretch of putative pseudouridine residues that are surrounded by bases with complementary base pairing with snoRNA H1, a snoRNA housed in a *Nop60B* intron, and U70 snoRNA, the human equivalent. The similarities to three pseudouridine residues within 18S rRNA catalyzed by Nop60B suggest that viral RNA could be a direct target of Nop60B. A silent mutation of putative pseudouridine nt9470 in E2 reduces SINV replication in insect and mammalian cells. Using Psi-seq, we confirmed that pseudouridylation is prevented at nt9470. Interestingly, mutating nt9470 also prevented pseudouridylation at nt9469, but not at site nt9468. These results suggest that preventing pseudouridine formation at one site can affect the formation of pseudouridine at a different site. The mechanism by which inhibition of pseudouridylation at nt9469 and nt9470 reduces viral replication remains unclear. Potential consequences include disrupted RNA-protein interactions with host or viral factors, altered RNA immunogenicity, or compromised RNA stability. We hypothesize that Nop60B and dyskerin are directly targeting the virus genome; however, our results do not exclude the possibility that differential modification of host RNA species plays a role in regulating viral replication, which has previously been reported with m^6^A (31). We have also shown the encapsidation of 18S rRNA in SINV particles (32, 33). Since H/ACA RNP complexes catalyze pseudouridine formation in 18S rRNA, which is essential for rRNA maturation (21), this suggests an additional mechanism by which Nop60B and dyskerin could enhance viral replication. Overall, these findings suggest a role for pseudouridine in SINV replication.

SINV infection influences *Nop60B* isoform production in whole flies. Both bacterial and viral infections are known to alter host isoform usage, resulting in downstream effects such as changes in protein stoichiometry (34, 35). *Nop60B* isoforms primarily differ in length of their 3′ UTRs, which may affect mRNA stability. Our ongoing work aims to understand the role of SINV-induced changes in *Nop60B* isoforms, focusing on their impact on Nop60B protein levels and localization. *Nop60B* is also interesting given that there are seven snoRNAs housed within the gene. The organization of the *Nop60B* gene structure suggests that its expression may impact the formation of other RNA modifications, raising the question of how different RNA modifications might influence one another. Differential splicing can influence the release of the snoRNAs at the 3′ end of *Nop60B,* some of which are annotated to be involved in methylation, while others remain classified as orphan snoRNAs with unknown functions. Moreover, there are seven different transcripts of *DKC1*, the gene encoding dyskerin. Future work is needed to determine how isoform expression may be affected by virus replication. Interestingly, when exposed to cellular stress, dyskerin forms small puncta in the cytoplasm (36), which correlate with the location of virus replication. Future work will investigate the localization of dyskerin in response to virus replication. Lastly, while our work focuses on Nop60B and dyskerin, a predicted ortholog in *Aedes aegypti* suggests potential conservation of function in mosquito hosts (37).

Pseudouridine RNA modifications were previously identified in SINV RNA through LC-MS/MS (10). Our study provides insight into the location of putative pseudouridine residues, which enables us to investigate their potential role in SINV replication. We mainly identified putative pseudouridine residues in the structural genes. The two most prominent hits from the Psi-seq results, nt7745 and nt7759, are located in a stem-loop immediately downstream of a translation enhancer known as the downstream loop (DLP), with the second stem-loop thought to structurally support the first (38, 39). Given the proximity to the DLP, we hypothesize that pseudouridine may aid in the translation of the structural proteins, which are required in higher amounts compared to nonstructural proteins, and future work will explore this potential regulatory role. In this study, we focused on investigating the specific putative pseudouridine residue that we hypothesize is a potential target of Nop60B. Since Nop60B can be detected in the cytoplasm, we suspect that the complex can assemble at the viral genome. However, this remains an open question. We found that a silent mutation in SINV at nt9470 reduces viral replication, which challenges the assumption that silent mutations are functionally neutral. Silent mutations have been shown to impact mRNA stability, translation, splicing, and protein function (40–42). We do not suspect that the UUU-to-UUC mutation is due to codon usage bias as UUC is the preferred codon for phenylalanine in *D. melanogaster (43*). Our results suggest that RNA modifications could be an additional explanation for phenotypes observed from synonymous mutations.

In summary, we present putative pseudouridine RNA modifications in SINV RNA derived from *Drosophila* cells. Additionally, we show that a catalytic putative pseudouridine synthase, Nop60B, is a proviral host factor. This work contributes to the limited understanding of pseudouridine in virus infection regulation. Future work will map putative pseudouridine residues in mosquito and mammalian cell lines using Psi-seq.

## MATERIALS AND METHODS

### Cell culture and virus production

Baby hamster kidney fibroblasts (BHK-21), human osteosarcoma U2OS, and human embryonic kidney epithelial (HEKT293T) cells were grown at 37°C under 5% CO_2_ in 1× MEM (Corning) supplemented with 10% heat-inactivated fetal bovine serum (FBS) (Corning), 1% each l-Glutamine (Corning), non-essential amino acids (Corning), and 1% antibiotic-antimycotic (Corning). JW18 cells are colonized with the endosymbiont *Wolbachia*. We generated a *Wolbachia*-free cell line, JW18-TET, using tetracycline. *D. melanogaster* S2R+ cells and JW18-TET cells were maintained in Schneider's Insect Media (Sigma), supplemented with 10% heat-inactivated FBS and 1% antibiotic-antimycotic (Corning). P0 SINV TE12 untagged and SINV TE12 Capsid-mCherry viruses were made by transfecting *in vitro* transcribed viral RNA into BHK-21 cells with Lipofectamine LTX (Sigma-Aldrich). The nt9470 mutant virus was generated with PrimeStar site-directed mutagenesis (Takara) of the SINV TE12 Capsid-mCherry cDNA clone. The mutation was confirmed by sequencing the E2 region in the P0 virus stock. P1 viruses for injections were generated by infecting BHK-21 cells with SINV TE12 P0. P1 viral supernatant was harvested and purified by centrifugation at 43,000 × *g* for 2.5 h over 27% wt/vol sucrose cushion in HNE buffer (150 mM NaCl, 20 mM HEPES, and 0.1 mM EDTA) and resuspended in 1× phosphate-buffered saline (PBS). Viral titers were determined through standard plaque assays on BHK-21 cells.

### Fly husbandry, genetic crosses, and virus injections

*Drosophila* fly stock OreR was used as the wild-type background in this study. We used a Transgenic RNAi Project fly line carrying a UAS-*Nop60B* short hairpin obtained from the Bloomington Drosophila Stock Center (BDSC stock no. 36595: y [1] sc[*] {v [1] sev [21]; P{y[+t7.7] v[+t1.8]= TRiP.GL00555}attP2). Both OreR and 36595 contain *Wolbachia* infections. To generate *Wolbachia*-free stocks, flies were reared on standard cornmeal agar containing tetracycline for three generations. The microbiome was repopulated by transferring treated flies into a bottle previously occupied by males from the same genetic background. Tetracycline-cleared TRiP 36595 was used for shRNA-targeted knockdown of *Nop60B* gene expression (all isoforms) by driving *Nop60B* shRNA expression using a heat shock inducible GAL4 driver (BDSC stock no. 2077: w[*]; P{w[=mC]=GAL4-Hsp70.PB}2). Fly stocks were maintained at 22°C unless stated otherwise. For injection experiments, female, unmated 2- to 3-day-old flies were anesthetized with CO_2_ and injected with 50 nL of P1 SINV TE12 (10^10^ PFU/mL) or PBS into the thorax with a glass capillary needle. Flies were collected 2 days post-injections and flash frozen in liquid nitrogen and stored at −80°C for downstream processing.

### qRT-PCR

RNA was extracted from cells and whole flies using TRIzol (Sigma Aldrich) reagent protocol. cDNA was generated using MMulV Reverse Transcriptase (New England Biolab) with random hexamer primers (Integrated DNA Technologies). Samples were DNase treated with RQ1 RNase-free DNase (Promega) following the manufacturer’s protocol. Quantitative RT-PCR was performed with the SensiFAST SYBR Hi-ROX Kit (Bioline) and gene-specific primers (Table S2). Assays were run on the Applied Bioscience StepOnePlus qRT-PCR machine (Life Technologies). Gene expression was normalized to either Actin or Rpl32 primers using the Livak method. To detect *Nop60B* isoforms, we ordered gene blocks to serve as positive controls and for generating standard curves for RB, RF, RG, and all isoforms. We ordered isoform-specific primer sets and confirmed that they were within 5% efficiency of one another. Isoform qRT-PCR assays were run on a CFX Opus Real-Time PCR Instrument (Bio-Rad).

### Temperature gradient knockdown of *Nop60B*

We crossed TRiP line 36595-TET to a GAL4 HSP70 heat shock driver, which produces GAL4 in the presence of heat. Given that this driver is leaky (GAL4 production even at 22°C), we reared flies at 18°C, 22°C, and 25°C. Crosses were set up so that progeny would emerge at the same time. Furthermore, we reared 36595-TET in the absence of the driver at 18°C, 22°C, and 25°C. We collected flies and age-matched them for 2–3 days at 22°C prior to injections. Two days post-injection, flies were collected, flash-frozen in liquid nitrogen, and stored at −80°C prior to processing for qRT-PCR. *Nop60B* specific primers targeting all isoforms, E1 primers targeting SINV, and RpL32 primers for the host were used to calculate dCT values. We combined dCT data from each temperature to perform linear regressions.

### *Nop60B* knockdown in cell culture

Knockdown of Nop60B expression was achieved in JW18-TET cells using dsRNA targeting all isoforms of *Nop60B*. We generated dsRNA templates by PCR from Nop60B overexpression construct DNA described below and on nanoluciferase (nLuc) DNA. We generated primers for these templates containing the T7 promoter sequence at the 5′ end of both primers (Table S1). PCR products were purified and used to generate dsRNA with the T7 MEGAscript Kit (Invitrogen). dsRNA was purified using the RNeasy Mini Kit (Qiagen). We incubated cells with dsRNA (2 µg) in serum-free media for 30 min at room temperature. After incubation, 10% FBS was added to each well. Knockdown efficiency was assessed 2 days post-dsRNA introduction using qRT-PCR.

### Nop60B and DKC1 overexpression in cell culture

*D. melanogaster Nop60B* coding region was cloned into the pAFW expression vector (1111) (Gateway Vector Resources, DGRC), downstream and in-frame with a 3× HA-tag (N-terminus) using NEBuilder HiFi DNA Assembly. The human dyskerin coding region with a 3× HA-tag (C-terminus) (DGRC Stock 1666059) was cloned into a pCMV vector using NEBuilder HiFi DNA Assembly. Both HA-Nop60B and dyskerin-HA plasmids were used to generate catalytic mutants (HA-Nop60B D123S and dyskerin-HA D125S) with PrimeStar site-directed mutagenesis (Takara). Control vectors are pAWF and pCMV vectors with no inserts. Plasmids were transfected into *Drosophila* or HEK293T cells using FuGENE Transfection Reagent (Promega) according to the manufacturer's protocol. Expression of HA-tagged constructs was confirmed by western blot using an anti-HA monoclonal antibody (3724, Cell Signaling, 1:1,000 dilution in 2% milk in 1× TBS + 1% Tween-20). Anti-β-actin antibody was used as a cellular loading control (4967, Cell Signaling, 1:1,000 dilution in 2% milk in 1× TBS + 1% Tween-20). Dyskerin detection through western blot was performed with an anti-dyskerin monoclonal antibody (53234, Cell Signaling, 1:1,000 dilution in 2% milk in 1× PBS + 1% Tween-20). Goat anti-rabbit 750 (ThermoFisher) was used as the secondary antibody. PVDF membranes were processed by one of the two methods to visualize proteins: (i) membranes were cut into sections to separately detect HA-tagged constructs and actin with their respective antibodies to enhance signal for each protein, or (ii) membranes were probed for dyskerin, then treated with stripping solution (100 mM Glycine, 0.1% NP-40, and 1% SDS, pH 2) to remove the primary antibody before reprobing with the anti-actin antibody. Methods are indicated in the figure legends.

### Nuclear and cytoplasmic fractionation

S2R+ cells were transfected with HA-empty or HA-Nop60B. Forty-eight hours post-transfection, the cells were infected with SINV TE12 mCherry at an MOI of 5. Forty-eight hours post-infection, the cells were resuspended in 50 µl of PBS, and a portion was set aside for whole-cell lysate samples, which were lysed in RIPA buffer. We added 0.1% hypotonic buffer and incubated for 3 min. We used a dounce homogenizer (15 times slowly) to lyse the cells. We centrifuged at 1,000 rcf for 5 min. The supernatant was transferred to a new tube. The pellet (nuclear fraction) was resuspended in 0.3% isotonic buffer for 3 min on ice. The resulting fractions were used in western blot. Anti-β-actin antibody was used to detect the cytoplasmic fraction (4967, Cell Signaling, 1:1,000 dilution in 2% milk in 1× TBS + 1% Tween-20). Anti-Histone H3 was used to confirm separation of the nuclear fraction (9715, Cell Signaling, 1:1,000 in 2% Milk in 1× TBS + 1% Tween-20).

### snoRNA quantitative RT-PCR

S2R+ cells were infected with SINV TE12 at an MOI of 5 and collected 48 h post-infection. We followed the nuclear and cytoplasmic fractionation protocol and split samples in half for RNA and protein extractions. Western blots were performed as described in the Nuclear and Cytoplasmic Fractionation section. RNA was extracted from the fractions using TRIzol reagent. We ordered a DNA construct of snoRNA H1 downstream of a T7 promoter sequence from TWIST Bioscience. We generated an *in vitro* transcribed snoRNA H1 with the MEGAscript T7 Transcription Kit (ThermoFisher). RNA from the *in vitro* transcription was cleaned up using the RNeasy Mini Kit (Qiagen) following the manufacturer’s protocol. The samples and IVT were converted to cDNA with a specific primer containing a hairpin loop as previously described (44) and using MulV Reverse Transcriptase (NEB). We designed a forward primer targeting snoRNA H1 and universal reverse qPCR primer that targets the specific primer added to snoRNA H1 during the RT step (44). We ran each sample in duplicate alongside a standard curve. RNA copies were determined through the standard curve.

### Live-cell imaging

Live-cell imaging was performed using Incucyte S3 Live-Cell Analysis System (Sartorius Corporation). Vertebrate and insect cells were plated in well plates at different confluencies depending on experiment (Transfections/Infections 50%; Infections only 80%). Infections were conducted with SINV TE12 Capsid mCherry with MOIs listed in main text and figure legends. Four images were taken per well. Mean total red fluorescence area (µm²/Image) (total virus area [µm²/Image]) was calculated over time. The phase image channel was used to track cell confluency. In experiments using JW18-TET cells, which contain a GFP-Jupiter fusion protein (45), we captured total green fluorescence area (µm²/Image) to track cell confluency. For graphs, we took the mean total red fluorescence area (µm²/Image)/ mean total green fluorescence area (µm²/Image) and plotted virus area/cell area.

### Statistical analyses

Statistical analyses were conducted using GraphPad Prism 10 (GraphPad Software Inc., San Diego, CA).

### Psi-seq virus infections

JW18-TET cells were infected with SINV TE12 or SINV TE12 nt9470 at an MOI of 10 for 2 h. The inoculum was removed, and cells were washed with PBS. Infections were harvested 5 days post-infection, and RNA was isolated using TRIzol reagent following the manufacturer’s recommendations.

### *In vitro* transcription of RNA spike-in

We generated an RNA spike-in containing a single pseudouridine residue at position 43 to add to each sample. A gBLOCK gene fragment was ordered from IDT with the sequence: GGGAGGCGAGAACACACCACAACGAAAACGAGCAAAACCCGGTACGCAACACAAAAGCGAACAACGCGAAAAAGGACACCGAAGCGGAAGCAAAGACAACCAACAGAAAACAACCGCAAACAAACGGGACCAGACAACGCACCAGCAAAA (sequence excludes the T7 promoter). *In vitro* transcription was performed using the MEGAscript T7 Transcription Kit (Invitrogen). GTP, CTP, ATP, and Psi-TP (Trilink Biotechnologies) were used at a concentration of 75 nmol each. The *in vitro* transcribed RNA was cleaned up using the RNeasy Mini Kit (Qiagen).

### CMCT treatment and RNA-seq library preparation

rRNAs and other uncapped RNA species were depleted from samples using Terminator 5′-phosphate-dependent exonuclease (Lucigen) according to the manufacturer’s protocol. Spike-in RNA was added to each sample prior to splitting the sample into two for CMCT or mock treatment. CMC treatment was performed as described (46, 47). RNA was resuspended in 30 µL 200 mM CMCT in BEU buffer (50 mM bicine, pH 8.3, 4 mM EDTA, and 7 M urea) or in 30 µL of BEU buffer only for 1 h at 37°C with 300 rpm rotation. The reaction was stopped with 100 µL of 0.3 M sodium acetate and 0.1 mM EDTA (pH 5.6) (Buffer A). An ethanol precipitation and washes were performed to pellet the RNA. The RNA pellet was dissolved in 40 µL of 50 mM sodium bicarbonate, pH 10.4, and incubated at 37°C for 4 h. 100 µL of Buffer A was added to the reaction. RNA was precipitated, washed, and dissolved in RNase-free water for library preparation.

Illumina sequencing libraries were prepared as described in reference 26. The single-stranded cDNA product was amplified for 10 cycles in a PCR reaction. Libraries were sequenced on Illumina NextSeq 2000 and NextSeq 1000 with paired-end reads. The demultiplexing of the reads was performed using bcl2fastq, version 2.20.0. Sequencing data can be found within the NCBI BioProject PRJNA1208211.

### Read trimming and mapping

Reads were adapter-trimmed and quality-filtered using Trimmomatic ver. 0.38, setting the cutoff threshold for average base quality score at 20 over a window of three bases, excluding reads shorter than 20 bases post-trimming (parameters: LEADING:20 TRAILING:20 SLIDINGWINDOW:3:20 MINLEN:20) (48). Reads were mapped to a genome reference prepared by combining the SINV genome sequence and RNA_spike_174 control molecule (26) using bowtie2 version 2.4.2 (49). Alignments from concordantly mapped read pairs were interpolated to define the inserts representing the reverse transcripts. For each insert, the reverse transcriptase termination site (5′ end of R2) was recorded.

### Detection of putative psi sites

Methods for calculating psi fold change and determining significant putative pseudouridine residues are described in reference 50. We used an FDR < 0.0005 in our study.

## SUPPLEMENTAL MATERIAL

Supplemental figures mbio.01329-25-s0001.pdf Fig. S1 to S6.

Tables S1 to S5 mbio.01329-25-s0002.xlsx Primers, *psi* sites, and data for psi sites.

Tables S6 to S8 mbio.01329-25-s0003.xlsx Data for *psi* sites.

ASM does not own the copyrights to Supplemental Material that may be linked to, or accessed through, an article. The authors have granted ASM a non-exclusive, world-wide license to publish the Supplemental Material files. Please contact the corresponding author directly for reuse.
