# Supplementary material for: Pseudouridine synthases are proviral factors for Sindbis virus in insect and mammalian cells
Source: mBio. 2025 May 27;16(7):e01329-25. doi: 10.1128/mbio.01329-25 (PMC12239590; doi:10.1128/mbio.01329-25)
Supplement: Supplemental figures — Fig. S1 to S6. [file mbio.01329-25-s0001.pdf]

Supplemental Figures For: Pseudouridine synthases are proviral factors for Sindbis virus in insect and mammalian cells

Authors: Nicole Stark, Ram Podicheti, Lauren Garcia, Adela Krenz, Douglas B Rusch, Irene L.G. Newton, R.W. Hardy

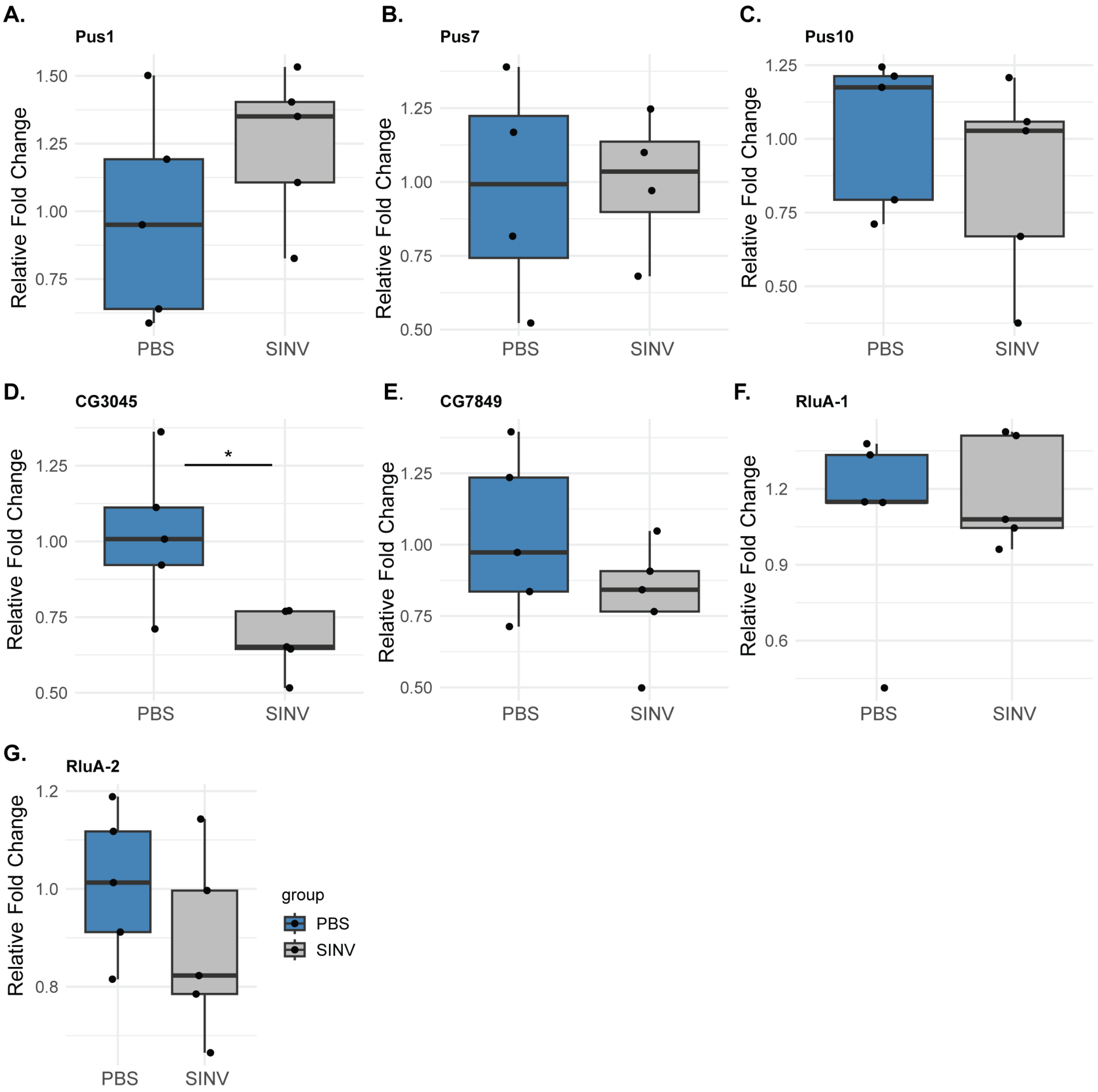

Figure S1. (A-G) Relative expression of 7/9 annotated pseudouridine synthases in *D melanogaster* measured two days after injection in adult female flies using qRT-PCR with primers designed to target all isoforms (Table S1). Each replicate represents an individual fly. D). CG3045 is significantly downregulated due to SINV infection (Unpaired, student's t-test p= 0.0170, t=3.002, df=8). Data normalized to *Actin*. We were unable to examine *CG34140* expression due to its dicistronic gene arrangement.

A.

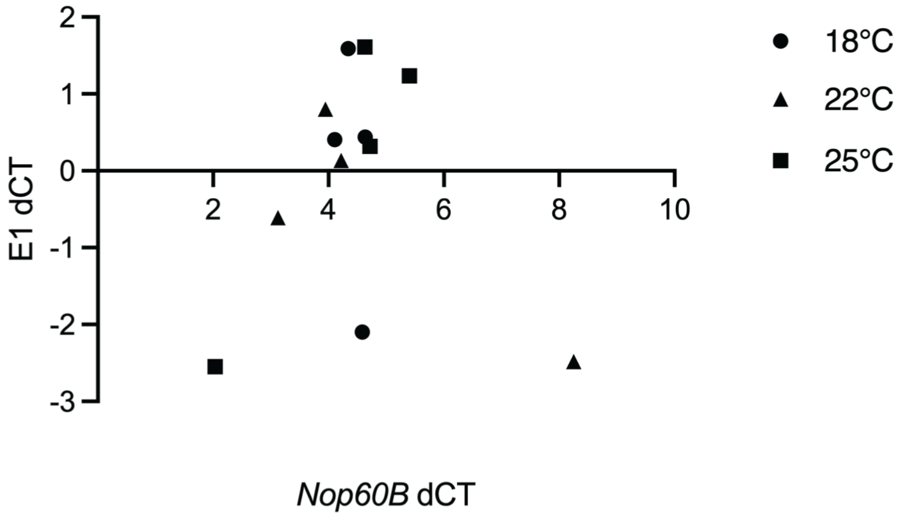

Figure S2. We reared the parental TRiP line (36595-TET) at 18°C, 22°C, and 25°C. Unmated, female flies were age matched for 2-3 days. Flies were injected with SINV TE12 at room temperature and infections proceeded for 48 hours before collection. We found no significant changes in E1 RNA levels due to rearing temperature. Furthermore, we found no significant correlation between *Nop60B* and E1, which is expected given the lack of variation in *Nop60B* RNA levels in this line (Pearson's r = -0.07477, p = 0.8174).

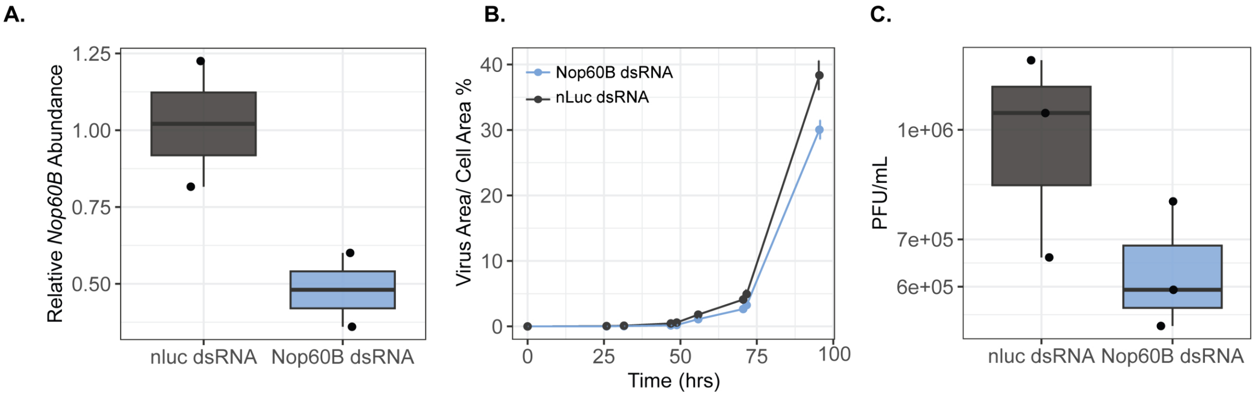

Figure S3: Knockdown of *Nop60B* in JW18-TET cells. A) Relative Nop60B Abundance in JW18-TET cells given non-targeting nLuc dsRNA compared to *Nop60B*-targeting dsRNA. Samples were collected two days-post dsRNA introduction. B) Virus Area/Cell Area % overtime. C) PFU/mL of samples in panel B taken four days post-infection. Statistical significance in panel B was assessed by Two-way ANOVA with Tukey's post hoc for multivariate analyses. Error bars represent standard error of mean (SEM) of independent experimental replicates.

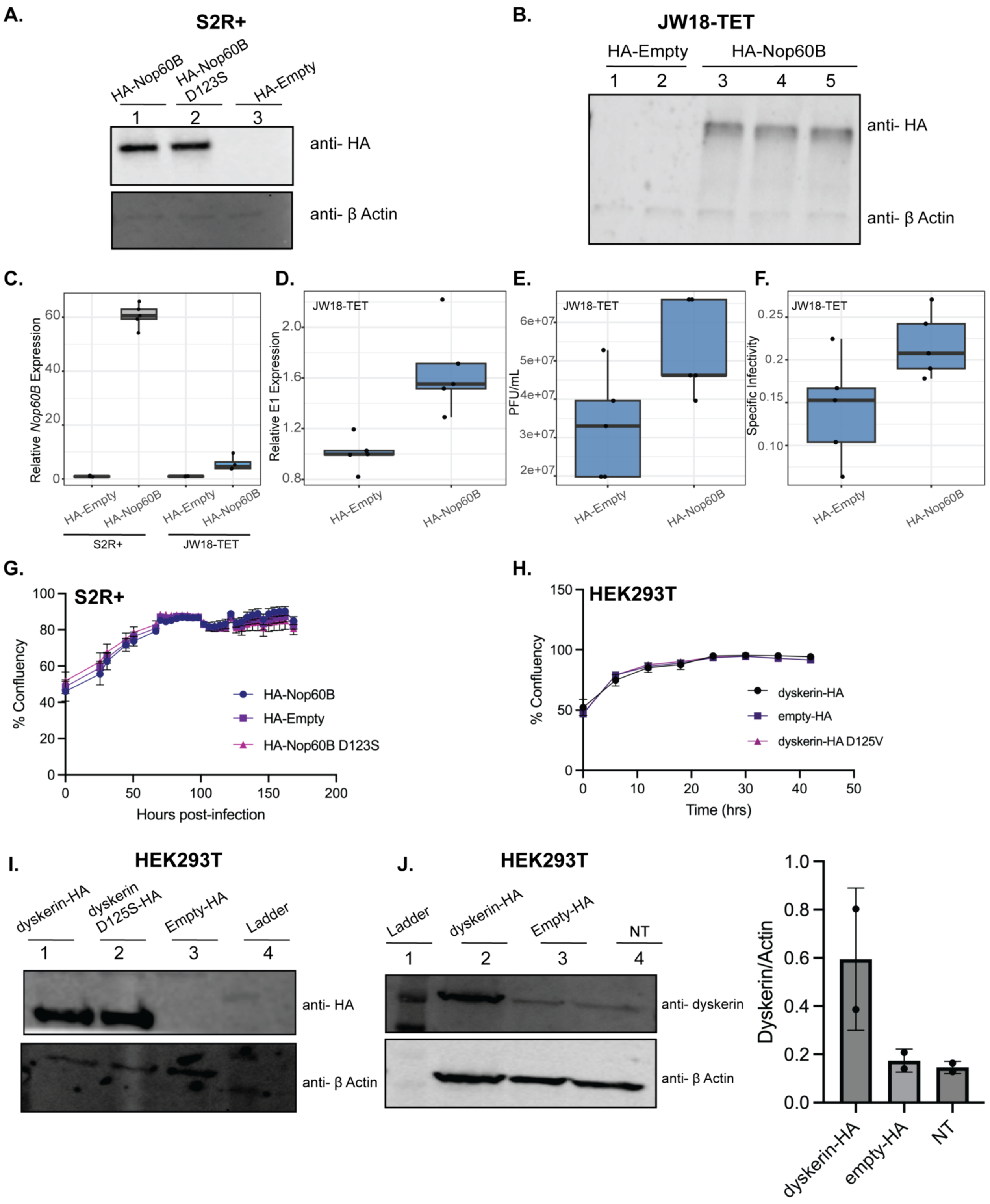

Figure S4. Western blot of HA-Empty, HA-Nop60B, and HA-Nop60B D123S overexpressed in A) S2R+ cells (membrane was stripped of anti-actin antibody before reprobing with anti-HA antibody) and B) JW18-TETs using anti-HA and anti- β Actin antibodies. C) Relative Nop60B expression in S2R+ and JW18-TET cells after over expression with HA-Empty or HA-Nop60B showing that HA-

Nop60B is highly expressed in S2R+ cells. When we overexpress HA-Nop60B in JW18-TET cells, we found a significant increase in D) Intracellular SINV RNA (p=0.0044), E) PFU/mL (p=0.0451), and F) Specific infectivity (p=0.0483) two days post-infection. G) Overexpression of HA-Nop60B and HA-Nop60b D123A has no impact on cell growth relative to HA-Empty. H) Overexpression of dyskerin-HA, empty-HA, and dyskerin-HA D125V has no impact on cell growth. I) Western blot of dyskerin-HA, dyskerin-HA D125S, and empty-HA overexpressed in HEK293T cells using anti-HA and anti- β Actin antibodies (membrane was cut to image separately). J) Representative western blot of dyskerin-HA, empty- HA, and a non-transfection (NT) control in HEK293T cells using an anti-dyskerin and anti- β Actin antibodies (membrane was stripped of anti-dyskerin antibody before reprobing with anti-actin antibody). Western quantified in ImageJ by generating histograms for each band and determining the area under the curve.

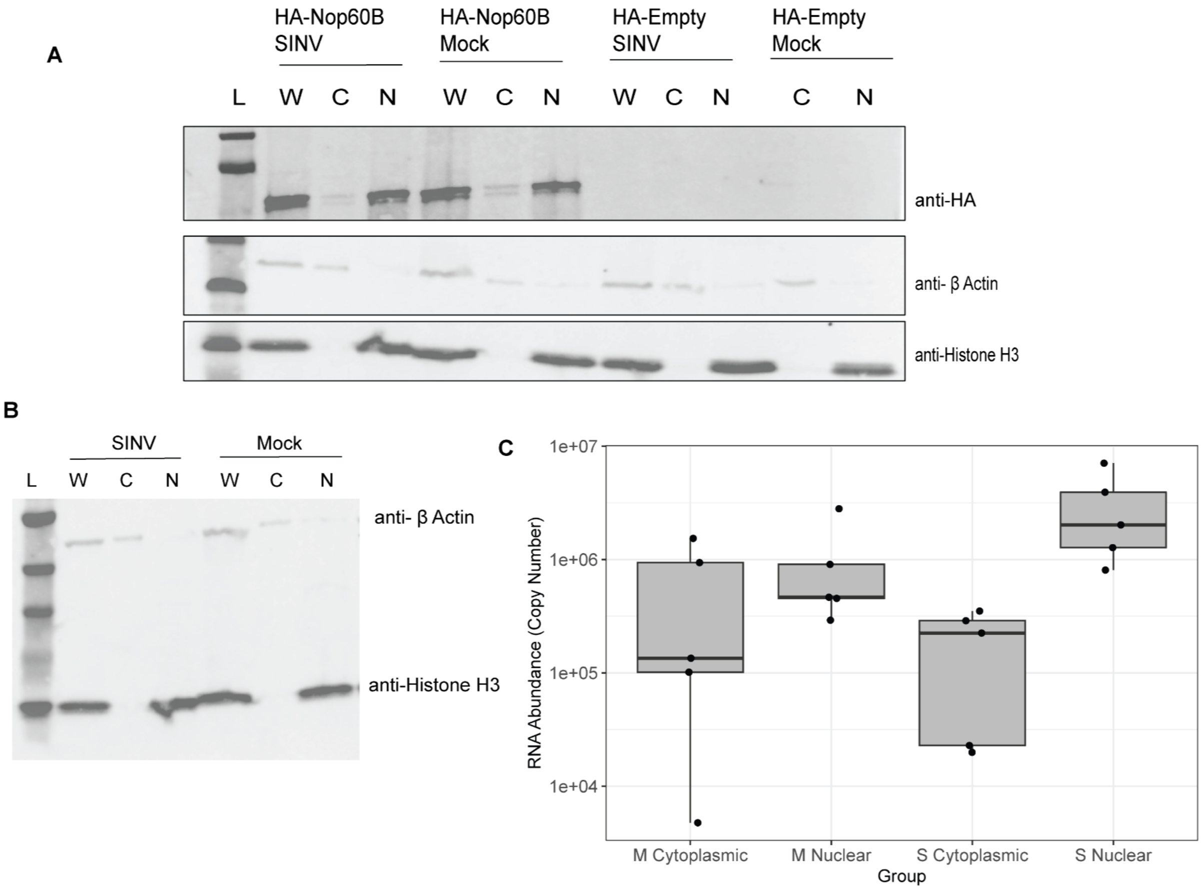

Figure S5. A) Western blots using anti-HA, anti- β Actin, and anti- Histone H3 antibodies on whole cell lysate, cytoplasmic, and nuclear fractions after mock and SINV infections in S2R+ cells transfected with HA-Nop60B or HA-Empty (MOI=5). B) Fractionation protocol done in mock and SINV infected S2R+ cells to look at endogenous snoRNA H1 localization in the cytoplasmic and nuclear fractions. C) RNA Abundance of snoRNA H1 following fractionation protocol in mock and SINV infected cells (MOI= 5) (M= Mock, S= SINV).

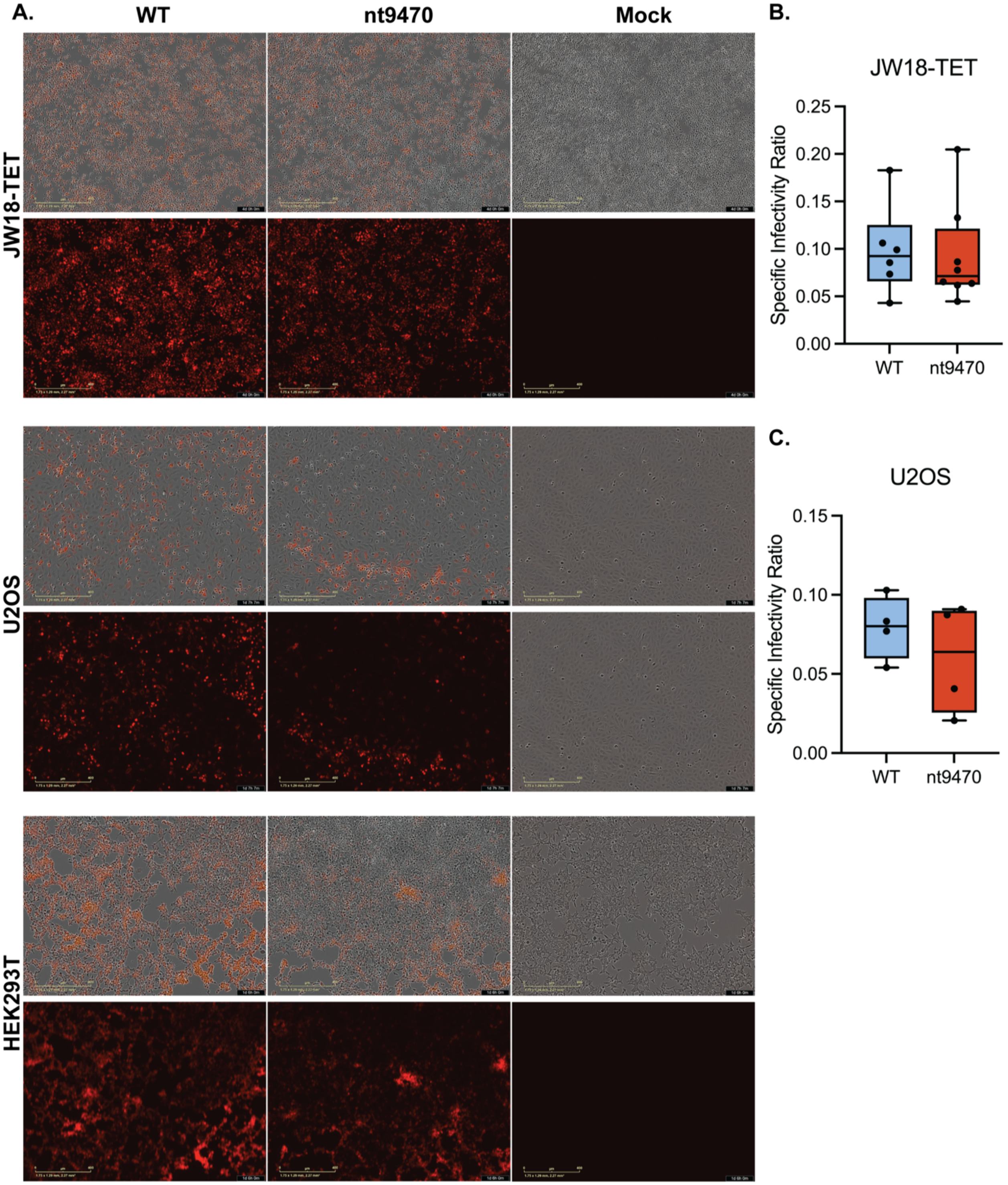

Figure S6. A) Representative images of SINV TE12 WT mCherry and SINV TE12 nt9470 mCherry in JW18-TET, U2OS, and HEK293T cells. Scale bar = 400 um. Specific infectivity in WT and nt9470 derived from B) JW18-TET and C) U2OS cells.
